# Identification of TRPV1-Inhibitory Peptides from *Takifugu fasciatus* Skin Hydrolysate and Their Skin-Soothing Mechanisms

**DOI:** 10.3390/md23050196

**Published:** 2025-04-29

**Authors:** Haiyan Tang, Bei Chen, Dong Zhang, Ruowen Wu, Kun Qiao, Kang Chen, Yongchang Su, Shuilin Cai, Min Xu, Shuji Liu, Zhiyu Liu

**Affiliations:** 1 College of Food Sciences & Technology, Shanghai Ocean University, Shanghai 201306, China; 2 Key Laboratory of Cultivation and High-Value Utilization of Marine Organisms in Fujian Province, National Research and Development Center for Marine Fish Processing, Fisheries Research Institute of Fujian, Xiamen 361013, China; 3 College of Chemical Engineering, Hua Qiao University, Xiamen 362021, China; 4 Mimask (Xiamen) Bio-Tech Co., Ltd., Xiamen 361000, China

**Keywords:** *Takifugu fasciatus*, TRPV1-inhibitory peptide, inflammatory response, bioactive peptide, NF-κB signaling, molecular docking network pharmacology

## Abstract

Skin sensitivity is increasingly prevalent, necessitating new therapeutic agents. This study screened multifunctional peptides from *Takifugu fasciatus* skin for transient receptor potential vanilloid 1 (TRPV1)-inhibitory and anti-inflammatory activities and investigated their mechanisms in alleviating sensitive skin (SS). A low-molecular-weight hydrolysate was prepared through enzymatic hydrolysis of *T. fasciatus* skin, followed by ultrafiltration, with subsequent peptide identification performed using nano-HPLC-MS/MS and molecular docking-based virtual screening. Among 20 TRPV1-antagonistic peptides (TFTIPs), QFF (T10), LDIF (T14), and FFR (T18) exhibited potent anti-inflammatory effects in (lipopolysaccharide) LPS-induced RAW 264.7 macrophages. T14 showed the strongest TRPV1 inhibition; T14 (200 μM) inhibited Ca^2^⁺ in capsaicin-stimulated HaCaT cells by 73.1% and showed stable binding in molecular docking, warranting further analysis. Mechanistic studies revealed that T14 suppressed NF-κB signaling by downregulating p65 protein expression, thereby reducing pro-inflammatory cytokine secretion (G-CSF, GM-CSF, ICAM-1, IL-6, TNF-α) in RAW 264.7 cells. Additionally, T14 (400 μM) inhibited ET-1 in LPS-stimulated endothelial cells by 75.0%; ICAM-1 reached 49.0%. Network pharmacology predicted STAT3, MAPK3, SPHK1, and CTSB as key targets mediating T14’s effects. These study findings suggest that T14 may be a promising candidate for skincare applications targeting SS.

## 1. Introduction

In recent years, the prevalence of sensitive skin (SS) has increased significantly because of environmental changes, work-related stress, and other multifactorial influences. SS is characterized by a hyperreactive skin state that occurs under physiological or pathological conditions, primarily affecting the facial region. Upon exposure to chemical or psychological stimuli, individuals experience subjective symptoms such as burning, stinging, itching, and tightness, which may be accompanied by objective signs such as erythema, scaling, and telangiectasia [1]. Epidemiological studies indicate that SS predominantly affects women, with reported prevalence rates of 36.1% in Chinese women [1], 60–70% in American women [2], 51% in British women [3], 25.01% in Russian women [4], and 36.7% in Indian women [5].

The pathogenesis of SS is complex and involves compromised skin barrier function, dysregulated neurovascular hyperactivity, and immune-inflammatory activation. Skin barrier dysfunction activates transient receptor potential vanilloid 1 (TRPV1). TRPV1 activation induces action potentials, leading to excessive Ca^2^⁺ influx [6], which mediates hallmark symptoms such as burning, stinging, and pruritus [7]. At the same time, TRPV1 stimulates keratinocytes and mast cells to release neuropeptides (e.g., substance P) [8] and pro-inflammatory cytokines [9], molecules that activate immune cells (e.g., T lymphocytes) and amplify inflammation [10]. In addition, TRPV1 interacts with vascular access. For example, it works synergistically with endothelin-1 (ET-1) to promote vascular dysfunction [11], upregulate intercellular adhesion molecule-1 (ICAM-1), and perpetuate inflammation [12].

Given these mechanisms, targeting TRPV1 inhibition presents a promising strategy for SS relief. However, current TRPV1 antagonists have limitations, including potential cytotoxicity and instability, highlighting the need for safer, naturally derived alternatives. Bioactive peptides derived from aquatic organisms such as fishes have shown potential as anti-inflammatory and skin-protective agents. Iosageanu et al. [13] demonstrated that bioactive peptides isolated from silver carp (*Hypophthalmichthys molitrix*) fish bones suppressed excessive reactive oxygen species production in ultraviolet B-irradiated skin cells, elevated the activity of antioxidant enzymes such as superoxide dismutase, and significantly reduced the release of pro-inflammatory cytokines by inhibiting the nuclear factor kappa-B (NF-κB) and mitogen-activated protein kinase (MAPK) signaling pathways. Xiong et al. [14] demonstrated that tilapia collagen peptide complex TY001 significantly accelerated the wound closure rate and improved the tissue regeneration quality in diabetic mice through three distinct mechanisms: suppressing inflammatory responses (evidenced by decreased tumor necrosis factor (TNF)-α and Interleukin (IL)-6 levels); enhancing the antioxidant capacity (indicated by elevated superoxide dismutase activity); and stimulating angiogenesis (mediated by upregulation of vascular endothelial growth factor (VEGF) and transforming growth factor-β (TGF-β) expression). Yu et al. [15] investigated the protective effects and mechanisms of collagen peptides derived from bigeye tuna (*Thunnus obesus*) skin and bone against ultraviolet B-induced skin photoaging. These peptides alleviated ultraviolet B-induced photoaging damage in keratinocyte (HaCaT) cells and dermal fibroblasts by suppressing the MAPK signaling pathway, thereby decreasing matrix metalloproteinase expression (MMP-1, MMP-9) while simultaneously activating the TGF-β/Smad pathway to enhance type I collagen synthesis. Maia Campos et al. [16] conducted a double-blind, placebo-controlled clinical trial demonstrating that daily supplementation with hydrolyzed fish cartilage extract (containing bioactive components such as collagen peptides and chondroitin sulfate) for 12 weeks significantly improved skin elasticity, stratum corneum hydration, and dermal density in the intervention group and markedly reduced wrinkle depth and skin roughness. *Takifugu fasciatus*, is a commercially significant species in Fujian province, China; the protein content of pufferfish skin accounts for more than 80% of the dry weight, which is a high-quality peptide extraction raw material [17]. However, its potential for TRPV1 inhibition and SS alleviation remains unexplored. Our previous research on *T. bimaculatus* collagen peptides demonstrated moisturizing and anti-inflammatory effects [17,18,19], prompting further investigation into *T. fasciatus* as a source of TRPV1-inhibitory peptides.

To address this gap, we aimed to identify TRPV1-inhibitory peptides from *T. fasciatus* skin hydrolysate and evaluate their potential to alleviate SS. We employed enzymatic hydrolysis, molecular docking-based virtual screening, and in vitro assays to identify and characterize bioactive peptides with dual TRPV1-inhibitory and anti-inflammatory activities. These findings provide a foundation for developing innovative skincare formulations targeting SS.

## 2. Results

### 2.1. Virtual Screening Based on the TRPV1 Receptor

Peptide sequences in the low-molecular weight *T. fasciatus* skin hydrolysate fraction were identified using nano-scale high-performance liquid chromatography-MS/MS. The −10 logp metric, which reflects the confidence level of spectral matches (where higher values indicate better matching reliability), was used to filter peptides with −10 logp ≥ 20 and ≤10 amino acid residues, yielding 190 candidate peptide sequences. These 190 peptides were subjected to molecular docking with the TRPV1 receptor protein (PDB: 8GFA). Based on grid score values and binding mode analysis, 20 hit peptides were prioritized (Table 1).

### 2.2. Inhibitory Effects of Hit Peptides on NO Production

**Figure 1:**
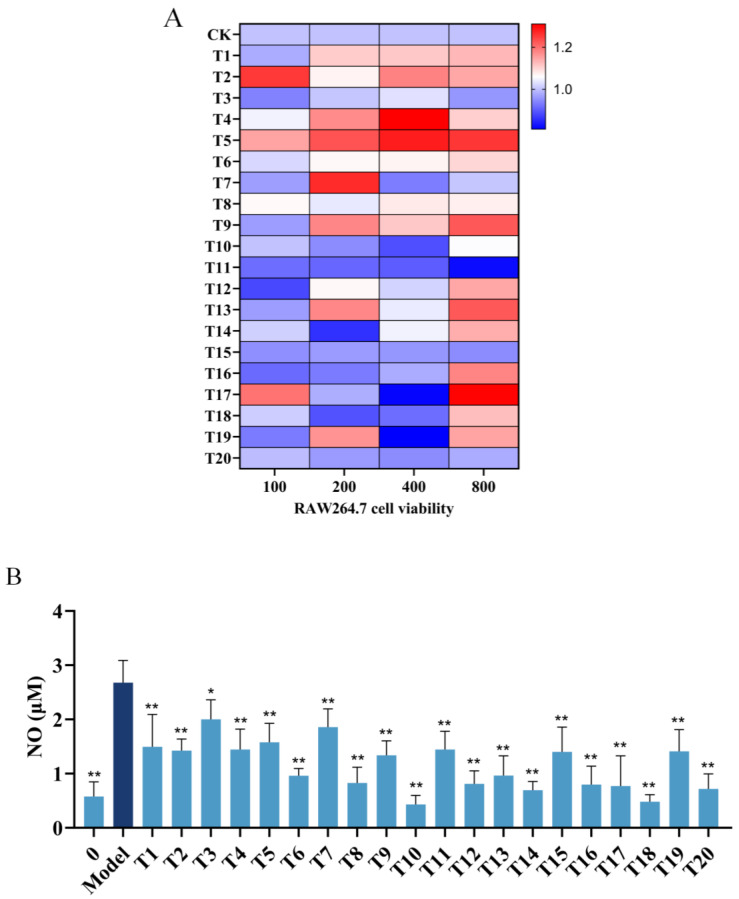
Effects of T1–T20 on NO production in lipopolysaccharide (LPS)-induced RAW 264.7 cells. Cells were pretreated with T1–T20 (200 μM) for 2 h, followed by the addition of LPS and co-incubation for 24 h. The NO concentration in the collected cell supernatant was measured using an NO assay kit. (**A**) Effect of T1–T20 on the viability of RAW 264.7 cells. (**B**) Inhibitory effect of T1–T20 on NO production. Data were analyzed using one-way analysis of variance followed by Dunnett’s multiple comparison tests. Means marked with “*” differ significantly from the model group (* *p* < 0.05, ** *p* < 0.01).

The cytotoxicity of 20 hit peptides toward RAW 264.7 cells was assessed in an MTS assay across a concentration range of 0–800 μM. As shown in Figure 1A, in most cases, the cells exhibited ≥90% cell viability following treatment with 800 μM peptides. Notably, peptides T17 and T19 reduced viability to 80% at 400 μM, whereas T5, T9, and T17 demonstrated proliferative effects at 800 μM. Based on these findings, 200 μM was selected as the optimal concentration for subsequent lipopolysaccharide (LPS)-induced inflammation experiments.

Nitric oxide (NO), a pro-inflammatory mediator linked to systemic inflammatory responses [20], was quantified using the Griess reagent assay to evaluate the anti-inflammatory potential of the 20 peptides. Compared with the LPS-treated control group, all peptides suppressed NO production (Figure 1B), with T10, T14, and T18 showing significant inhibition (*p* < 0.05). These three peptides were selected for further mechanistic studies.

### 2.3. Inhibitory Effects of Hit Peptides on Capsaicin (CAP)-Induced Ca^2+^ Influx in HaCaT Cells

**Figure 2:**
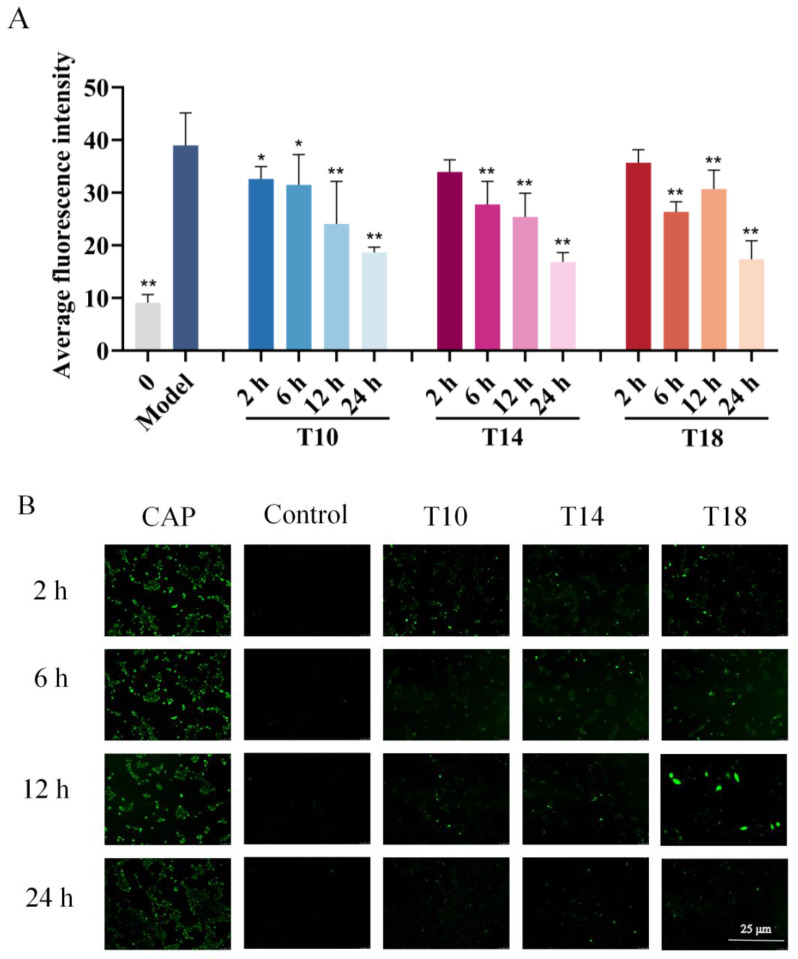
T10, T14, and T18 inhibit Ca^2^⁺ production in capsaicin (CAP)-induced keratinocyte (HaCaT) cells. Cells were pretreated with T10, T14, and T18 (200 μM) for 2, 6, 12, and 24 h, followed by incubation with Fluo-4AM for 1 h, washing three times with Hank’s Balanced Salt Solution, and treatment with CAP for 2 h. Fluorescence images were captured using an inverted fluorescence microscope. (**A**) Effects of T10, T14, and T18 on intracellular Ca^2^⁺ in CAP-induced HaCaT cells. (**B**) Representative fluorescence images of Ca^2^⁺ inhibition by T10, T14, and T18. Images were processed using ImageJ 1.8.0-265 (64-bit) software, and data were analyzed using one-way ANOVA followed by Dunnett’s multiple comparison tests. Mean values marked with “*” differ significantly from the model group (* *p* < 0.05, ** *p* < 0.01).

A CAP-induced HaCaT cell model was established to investigate TRPV1-mediated skin desensitization mechanisms, with intracellular Ca^2+^ influx as the primary indicator. As shown in Figure 2, CAP significantly elevated intracellular Ca^2+^ levels in HaCaT cells. Pretreatment with 200 μM of T10, T14, or T18 peptides markedly inhibited CAP-induced Ca^2+^ influx, demonstrating a time-dependent effect. Prolonged incubation (12–24 h) enhanced this inhibitory activity, with maximal suppression observed at 24 h. Quantitative analysis revealed the following inhibition rates of Ca^2^⁺ fluorescence intensity after 24 h treatment: T10: 66.8% ± 7.1%; T14: 73.1% ± 7.6%; T18: 72.4% ± 8.4%. T14 exhibited the strongest inhibitory effect on Ca^2+^ influx, making it the leading candidate for subsequent studies.

### 2.4. Molecular Docking of Candidate Polypeptides with TRPV1

**Figure 3:**
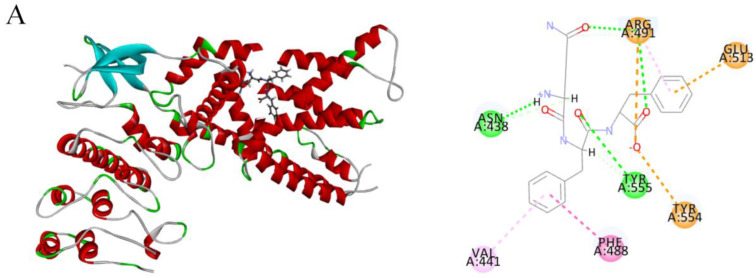

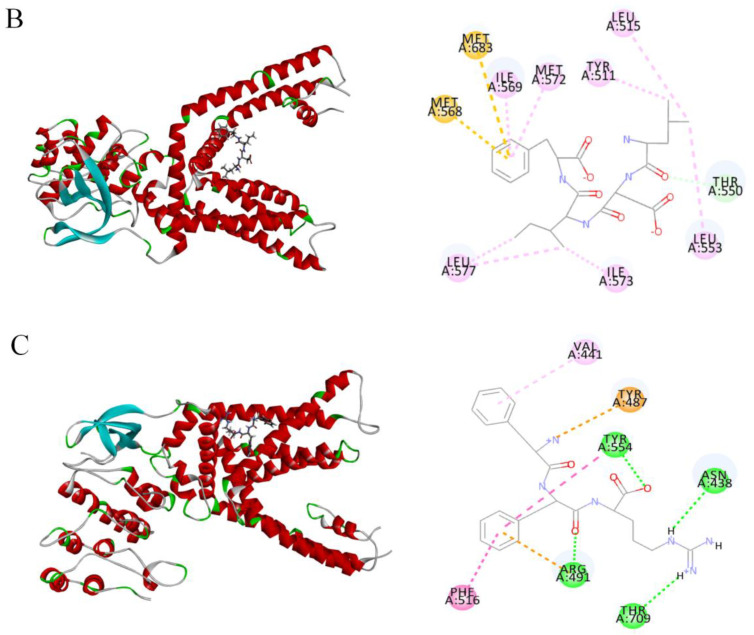
3D and 2D diagrams of molecular docking complex patterns of peptides with TRPV1. (**A**) T10-TRPV1 complex, (**B**) T14-TRPV1 complex, and (**C**) T18-TRPV1 complex. T10 interacted with ASN438, ARG491, TYR555, VAL441, ARG491, ARG491, GLU513, TYR554, and PHE488 of TRPV1. T14 interacted with THR550, MET572, LEU553, TYR511, LEU515, LEU553, LEU577, and ILE569 of TRPV1. T18 interacted with TYR554, TYR554, TYR554, ARG491, TYR554, VAL444, ARG491, TYR487, and PHE516 of TRPV1. Note: Green = hydrogen bonds; purple = hydrophobic interactions; orange = electrostatic interactions; yellow = disulfide bonds.

The active site of TRPV1 includes a vanilloid-binding pocket, a phospholipid-binding pocket, and other small molecule-binding pockets. As shown in the 3D molecular docking structure (Figure 3), T10, T14, and T18 were bound to the active cavity of TRPV1. The binding strength between small molecules and macromolecules is typically determined by changes in free energy during docking. Intermolecular forces, such as hydrogen bonds, electrostatic interactions, and hydrophobic interactions, significantly influence binding affinity and mode, often stabilizing complex formation [21].

As indicated in Table 2, T10 formed three hydrogen bonds, three hydrophobic interactions, and three electrostatic interactions with TRPV1 residues. T14 formed two hydrogen bonds, seven hydrophobic interactions, and two disulfide bonds, whereas T18 formed three hydrogen bonds, four hydrophobic interactions, and two electrostatic interactions with TRPV1 residues. These interactions contribute to the structural stability of the complexes. The root-mean-square deviation (RMSD) reflects changes in protein–ligand interactions between the crystal structures before and after docking. Lower RMSD values indicate that the docked ligand conformation closely matches the binding site of the target protein [22]. As shown in Table 2, the RMSD values for the docking of the three polypeptides with TRPV1 were T10: 3.27, T14: 2.96, and T18: 3.24. These low RMSD values suggest that the peptide conformations closely matched the TRPV1 binding sites. Based on the inhibition of Ca^2^⁺ levels and molecular docking analysis, T14 was identified as the most promising candidate for further experimental investigation.

### 2.5. Effects of T14 on Cytokine Expression Levels in RAW 264.7 Cells

**Figure 4:**
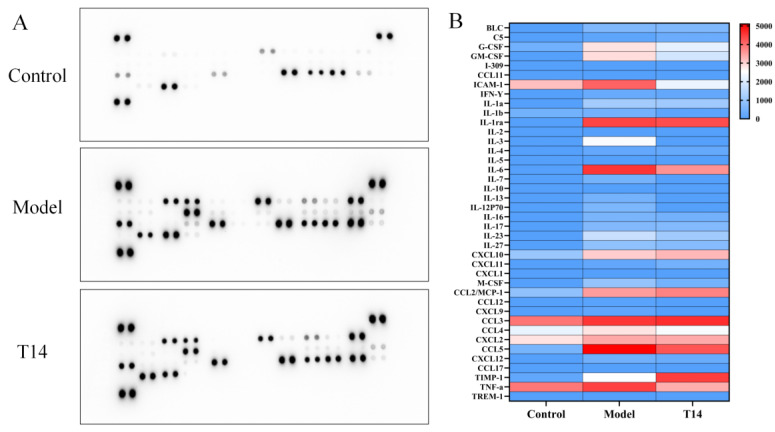
T14 suppresses cytokine expression in lipopolysaccharide (LPS)-induced RAW 264.7 cells. RAW 264.7 cells were pretreated with T14 (0, 200 μM) for 6 h, followed by co-incubation with LPS for 24 h. After treatment, cell culture supernatants were collected, and cytokine production was analyzed using the Proteome Profiler™ Antibody Arrays Mouse Cytokine Array Panel A kit. The pixel density of each spot in panel (**A**) was analyzed using ImageJ software to quantify cytokine expression levels. (**A**) Cytokine array of RAW2 64.7 cells. (**B**) Heat map of gene expression of 40 cytokines. Note: Each pair of points in (**A**) represents two parallels for each cytokine or chemokine.

Cytokines are small molecular proteins with diverse biological functions, synthesized and secreted by immune cells (e.g., lymphocytes and monocytes/macrophages) and certain non-immune cells (e.g., vascular endothelial cells and epidermal cells) in response to various stimuli [23]. The major cytokine families include ILs, interferons, TNF, colony-stimulating factors (CSFs), and chemokines [24]. As shown in Figure 4, T14 significantly inhibited LPS-induced expression of pro-inflammatory cytokines in RAW 264.7 cells. Compared with the normal group, the LPS-treated group exhibited significant upregulation in the expression levels of G-CSF, GM-CSF, ICAM-1, IL-3, IL-6, TNF-α, CCL3, CCL4, CXCL2, CCL5, and TIMP-1. Mild upregulation was also observed for M-CSF, IL-1α, IL-7, IL-13, IL-12P70, IL-16, IL-17, IL-23, and IL-27, although these changes were not significant. Following T14 treatment, the expression levels of G-CSF, GM-CSF, ICAM-1, IL-3, IL-6, IL-23, and TNF-α were significantly downregulated. A moderate downward trend was also observed for M-CSF, IL-1α, IL-7, IL-13, IL-12P70, IL-16, IL-17, IL-27, CCL3, CCL4, CXCL2, and CCL5. Notably, TIMP-1 expression was markedly upregulated.

### 2.6. T14 Inhibits the NF-κB Signaling Pathway in CAP-Induced HaCaT Cells

**Figure 5:**
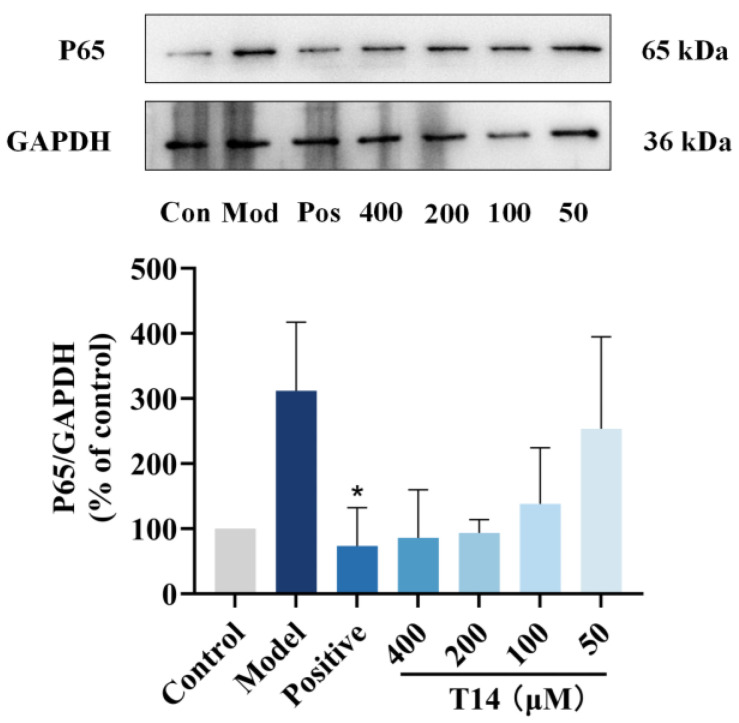
Effect of T14 on NF-κB translocation in lipopolysaccharide (LPS)-induced keratinocyte (HaCaT) cells. Western blot analysis was performed to assess the impact of T14 treatment on p65 expression in total cellular proteins. HaCaT cells were grown in 6-well plates, pretreated with varying concentrations of T14 for 6 h, and then co-incubated with CAP for 24 h. After treatment, cell lysates were collected and subjected to Western blotting using a p65-specific antibody. Protein band intensities were quantified using ImageJ software to determine changes in p65 expression levels. Data were analyzed using one-way ANOVA followed by Dunnett’s multiple comparison tests. Mean values marked with “*” differ significantly from the model group (*p* < 0.05).

NF-κB is a transcription factor that regulates the gene expression of growth factors, chemokines, cytokines, and cell adhesion molecules. Upon cellular stimulation, the NF-κB signaling pathway is activated, triggering inflammatory responses. To determine whether T14 suppresses p65 (a key NF-κB subunit) in CAP-induced HaCaT cells, Western blot analysis was performed. As shown in Figure 5, CAP induction significantly increased p65 levels in HaCaT cells. However, T14 treatment markedly reduced p65 expression, confirming that T14 inhibits the NF-κB pathway, thereby suppressing the expression of pro-inflammatory cytokines.

### 2.7. T14 Reduces Inflammatory Responses in HUVECs

**Figure 6:**
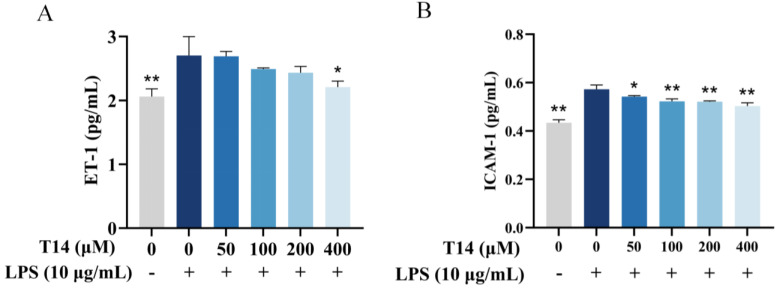
T14 inhibits ET-1 and ICAM-1 expression and secretion in lipopolysaccharide (LPS)-induced human umbilical vein epithelial cells (HUVECs). HUVECs were pretreated with varying concentrations of T14 (0, 50, 100, 200, 400 μM) for 6 h, followed by co-incubation with LPS for 24 h. After treatment, cell culture supernatants were collected, and the expression levels of ET-1 and ICAM-1 were quantified using specific ELISA kits. (**A**) Inhibitory effect of T14 on ET-1 expression. (**B**) Inhibitory effect of T14 on ICAM-1 expression. Data were analyzed using one-way ANOVA followed by Dunnett’s multiple comparison tests. Mean values marked with “*” differ significantly from the model group (* *p* < 0.05, ** *p* < 0.01).

When vascular endothelial cells are exposed to endogenous or exogenous stimuli, ET-1 secretion increases. Elevated ET-1 levels stimulate neutrophils and macrophages to release pro-inflammatory factors [25], whereas ICAM-1 expression surges, enhancing vascular permeability, causing skin erythema, and triggering vascular irritation [26]. As shown in Figure 6A, LPS stimulation (10 μg/mL, 24 h) increased ET-1 levels in human umbilical vein epithelial cells (HUVECs) from 2.05 to 2.7 pg/mL. However, pretreatment with T14 for 6 h, followed by LPS co-culture (24 h), suppressed ET-1 expression in a dose-dependent manner: At 100 μM T14, ET-1 decreased to 0.54 pg/mL (inhibition rate: 24.47%). At 400 μM T14, ET-1 levels decreased to 2.2 pg/mL (inhibition rate: 75%), demonstrating a significant difference compared to the LPS group.

As shown in Figure 6B, LPS stimulation elevated ICAM-1 expression from 0.43 to 0.57 pg/mL. T14 pretreatment similarly exhibited dose-dependent inhibition: at 50 μM T14, ICAM-1 decreased to 0.54 pg/mL (inhibition rate: 20.65%). At 400 μM T14, ICAM-1 levels fell to 0.50 pg/mL (inhibition rate: 48.96%), showing significant differences.

### 2.8. Network Pharmacological Analysis

#### 2.8.1. Prediction of T14 Action Targets

**Figure 7:**
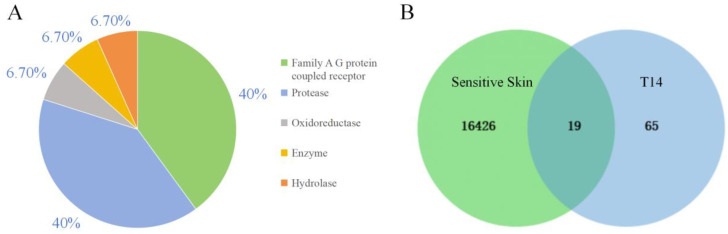
(**A**) Distribution of potential targets of T14 action, classified under family A G protein-coupled receptor, protease, oxidoreductase, enzyme, and hydrolase. (**B**) Venn diagram of T14 and sensitive skin (SS) intersection targets, showing 19 overlapping potential targets.

Using the Swiss Target Prediction platform, 84 potential targets of T14 were predicted in vivo, and their distribution is shown in Figure 7A. A total of 16,445 SS-related gene targets were identified from the GeneCards database. By integrating T14- and SS-related targets into Venn diagram software (Figure 7B), 19 shared potential targets were identified. These results suggest that T14 acts through these 19 shared targets to modulate the onset and progression of SS.

#### 2.8.2. PPI Network Construction

**Figure 8:**
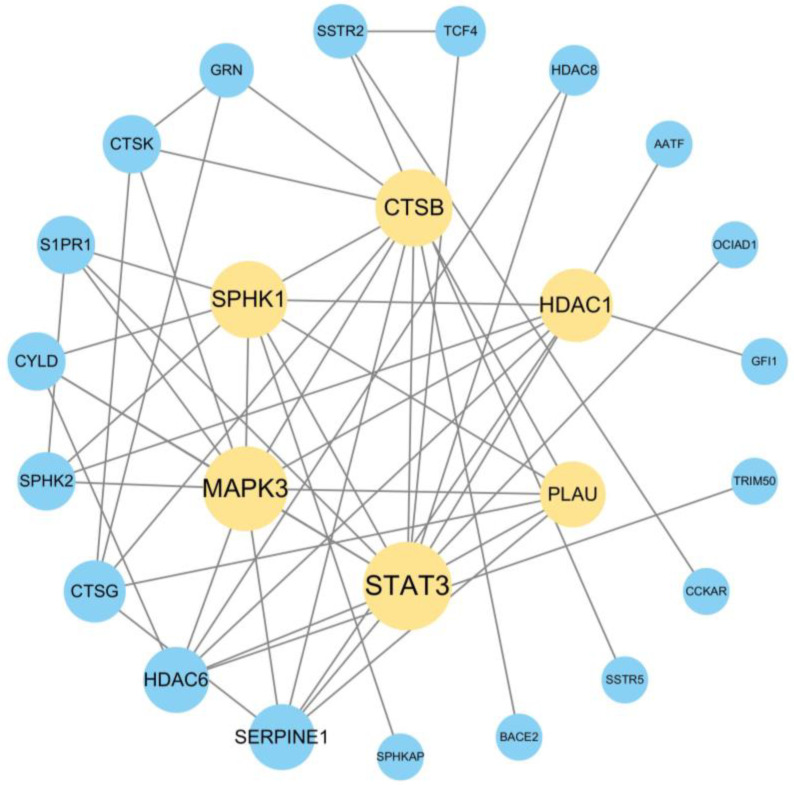
Protein–protein interaction network diagram of T14 action targets. The protein–protein interaction network consisted of 25 nodes (representing proteins) and 53 edges (representing interactions).

These 19 shared targets were uploaded to the STRING platform to generate a protein–protein interaction (PPI) network, which was further refined using Cytoscape 3.10.0 (Figure 8). The PPI network consisted of 25 nodes (representing proteins) and 53 edges (representing interactions). The node size was correlated with the degree value (a measure of connectivity). Targets with degree values exceeding the network average (8.48) were identified as core targets because they played a central role in the network [27]. Nine core targets were screened based on their degree rankings (Table 3), suggesting their critical involvement in the ability of T14 to modulate SS. Signal transducer and activator of transcription 3 (STAT3), MAPK3, sphingosine kinase 1 (SPHK1), and cathepsin B (CTSB) exhibited the largest nodes in the PPI network, suggesting that they play pivotal roles in the therapeutic effects of T14 on SS pathogenesis.

#### 2.8.3. KEGG Pathway Enrichment and GO Enrichment Analyses

**Figure 9:**
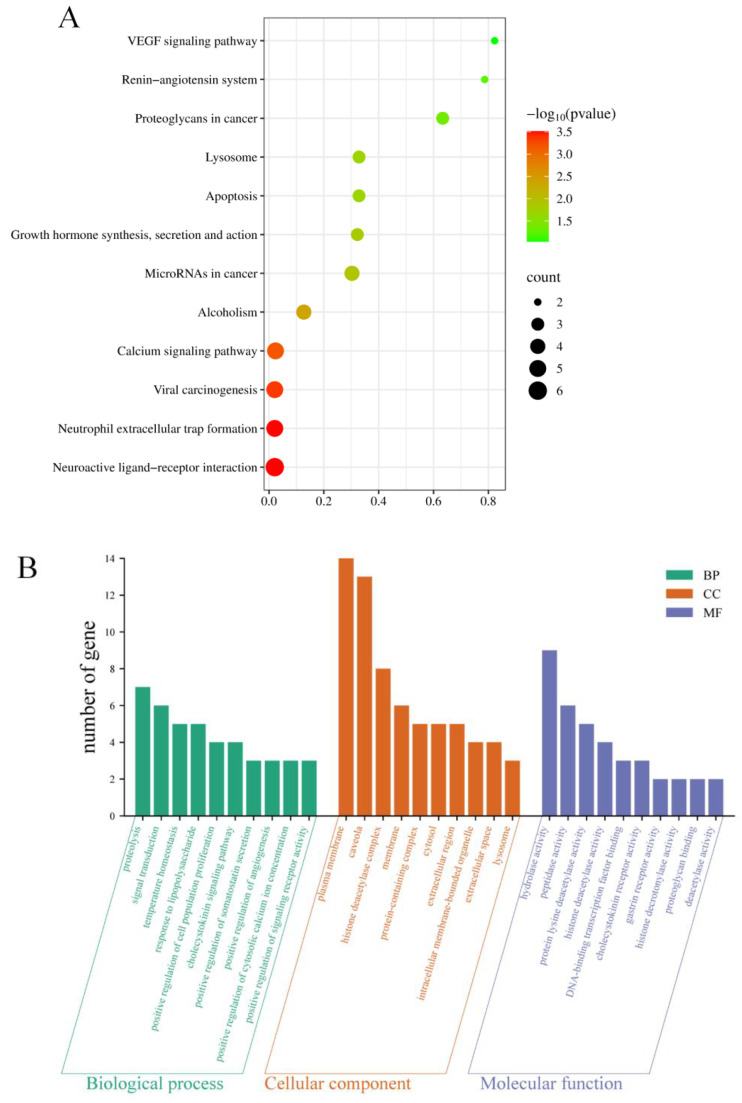
Biological information analysis of T14 and its targets in sensitive skin. (**A**) KEGG pathway enrichment analysis of 12 putative targets. (**B**) Gene ontology annotations, including BP, CC, and MF analyses. KEGG: Kyoto Encyclopedia of Genes and Genomes; BP: biological process; MF: molecular function; CC: cellular component.

To further evaluate the potential mechanisms of T14 in the prevention and treatment of SS, Kyoto Encyclopedia of Genes and Genomes (KEGG) pathway and Gene Ontology (GO) enrichment analyses were performed on 19 shared targets using the DAVID database. As shown in Figure 9A, KEGG pathway enrichment identified 12 significantly enriched signaling pathways (*p* < 0.05), including neuroactive ligand–receptor interactions, neutrophil extracellular trap formation, calcium signaling pathway, pathways in cancer, apoptosis, and the VEGF signaling pathway. GO analysis categorized enriched terms into biological processes, cellular components, and molecular functions. A total of 43 biological processes (BP), 19 cellular components (CC), and 26 molecular function (MF) terms were identified. The top 10 enriched terms (*p* < 0.05) in each category are shown in Figure 9B. Key BP terms included proteolysis, signal transduction, positive regulation of cell population proliferation, and positive regulation of angiogenesis.

## 3. Discussion

This study focused on peptides derived from *T. fasciatus* skin hydrolysate that specifically target the TRPV1 receptor. Virtual screening of the pufferfish peptide database identified 20 candidate polypeptides based on docking scores. Among these, T10, T14, and T18 were selected for further investigation, as they significantly inhibited NO production in RAW 264.7 cells at 200 μM. All three peptides reduced intracellular Ca^2+^ levels in CAP-treated HaCaT cells, suggesting their potential TRPV1 antagonistic activity.

The TRPV1 antagonist capsazepine competitively inhibits CAP binding by targeting the S3–S4 and S4–S5 transmembrane regions of TRPV1 [28]. T14 formed one hydrogen bond with THR550 (2.24 Å) and eight hydrophobic interactions with the residues MET572 (4.26 Å), LEU553 (5.27 Å), TYR511 (4.59 Å), LEU515 (5.32 Å), LEU577 (4.83 Å, 4.96 Å), and ILE569 (4.78 Å). Notably, T14 shares key residues with capsazepine, including TYR511 (S3 helix) and LEU553, ILE569, and MET572 (S3–S4/S4–S5 regions), stabilizing its binding via hydrophobic interactions. Molecular docking revealed that T14 occupies the TRPV1 active cavity in a spatial arrangement resembling that of vanilloid compounds, potentially explaining its potent inhibitory effects.

LPS stimulation significantly upregulated G-CSF and GM-CSF in RAW 264.7 cells, promoting granulocyte generation and activation. Activated granulocytes rapidly accumulate at inflammatory sites, exacerbating dysregulation of the inflammatory microenvironment [29]. T14 treatment markedly downregulated G-CSF and GM-CSF, thereby inhibiting granulocyte recruitment and activation as well as reducing inflammation intensity and tissue damage. IL-6 and TNF-α play key roles in activating downstream inflammatory pathways, particularly NF-κB. Upon activation, IκB kinase phosphorylates IκB, leading to its degradation. This releases NF-κB (p65 subunit), allowing its phosphorylation (p-p65) and the subsequent release of inflammatory mediators [30,31]. T14 significantly suppressed IL-6 and TNF-α expression, reducing NF-κB activation and inflammatory mediator release, which mitigates tissue damage and preserves tissue integrity.

LPS stimulation also upregulated CCL3, CCL4, CXCL2, and CCL5, which recruit monocytes, T cells, neutrophils, and natural killer cells to inflammatory sites [32,33]. T14 induced mild downregulation of these chemokines, potentially limiting immune cell infiltration and inflammation propagation. Although the reduction was modest, it may have attenuated the spread and duration of inflammation by reducing the immune cell burden at the site. LPS stimulation disrupts extracellular matrix homeostasis via MMP overexpression. During the inflammatory process, pro-inflammatory factors can enhance the expression of MMPs, promoting the apoptosis of dermal fibroblasts [18]. T14 upregulated TIMP-1, an endogenous MMP inhibitor, likely by suppressing pro-inflammatory pathways that drive MMP expression. Enhanced TIMP-1 activity stabilizes the extracellular matrix, prevents tissue degradation, and supports tissue repair [28].

SS is characterized by impaired barrier function, exacerbated inflammatory responses, and dysregulated neurovascular modulation. Emerging evidence indicates that STAT3 activation upregulates inflammation-associated gene expression during cellular stress and inflammatory responses, consequently exacerbating clinical manifestations of inflammation [34]. Furthermore, MAPK3 is an important member of the mitogen-activated protein kinase family, which is involved in cell proliferation, differentiation, apoptosis, and inflammatory response. Activation of MAPK3 initiates a pro-inflammatory cascade when the skin is exposed to external stimuli, leading to the occurrence of skin inflammation [35]. T14 may exert therapeutic potential through suppression of STAT3 and MAPK3 phosphorylation, effectively attenuating inflammatory mediator release and ameliorating cutaneous inflammatory responses.

BP terms enriched by T14, such as proteolysis, signal transduction, positive regulation of cell population proliferation, and angiogenesis, collectively contribute to skin repair. Proteolysis facilitates the clearance of abnormal proteins, creating conditions for normal skin cell metabolism and renewal, whereas signal transduction modulates cellular responses to stimuli and maintains skin homeostasis [36]. Enhanced cell proliferation supports skin regeneration and barrier restoration, and regulated angiogenesis improves blood circulation, ensuring adequate nutrient and oxygen supply for repair [37]. Among the 12 KEGG-enriched pathways, T14 may alleviate neuro-hyperreactivity (e.g., stinging and itching) by modulating neuroactive ligand–receptor interactions, reducing inflammation (e.g., erythema) via neutrophil extracellular trap formation, and restoring skin cell function (proliferation, differentiation, barrier integrity) through calcium signaling [38]. The normalization of apoptotic pathways facilitates the clearance of damaged or senescent cells, promoting healthy cellular turnover. Notably, TRPV1 activation in keratinocytes triggers calcium influx and apoptosis, as demonstrated by Bodó et al. [39]. Dysregulated apoptosis in SS—whether through insufficient clearance of inflammatory immune cells or excessive keratinocyte death—perpetuates inflammation via mediators such as histamine and TNF-α, exacerbating SS symptoms [40,41]. Furthermore, the suppression of ET-1 and ICAM-1 (linked to VEGF signaling) by T14 suggests its role in mitigating vascular hyperpermeability and inflammation [42,43]. By modulating multiple targets and pathways, T14 effectively alleviates SS symptoms while restoring structural and functional integrity.

While this investigation provides novel insights into T14’s therapeutic potential, several limitations warrant consideration. First, although T14 demonstrated strong TRPV1 inhibition and anti-inflammatory effects in vitro, its efficacy in vivo remains to be determined. Future studies are needed to evaluate its stability, bioavailability, and pharmacokinetics in animal models of SS. Additionally, although TRPV1 inhibition was associated with cytokine suppression, the potential involvement of alternative pathways, such as neuropeptide release, requires further investigation. Finally, the impact of T14 on non-target cell types, particularly sensory neurons, and immune cells, should be explored to ensure specificity and minimize off-target effects.

## 4. Materials and Methods

### 4.1. Preparation of Low-Molecular Weight T. fasciatus Skin Hydrolysate

Fresh *T. fasciatus* skin (Zhangpu, Zhangzhou, China) was diced using a meat mincer, freeze-dried to remove moisture, and stored in vacuum-sealed packaging. The lyophilized skin was immersed in 9% NaCl solution (1:30 *w*/*v*) under continuous stirring for 24 h, with solution renewal every 12 h. Following soaking, the skin was rinsed three times with distilled water and filtered through a nylon mesh to remove non-collagenous proteins. Enzymatic hydrolysis was performed as described by Zhang [19] using alkaline protease (B8360, Solarbio, Beijing, China) under optimized conditions: solid-to-liquid ratio of 1:10 (*w*/*v*), temperature 50 °C, enzyme dosage 8000 U/g, pH 9.0, and hydrolysis duration 4 h. The reaction was terminated by heating at 100 °C for 10 min. The hydrolysate was sequentially processed via ceramic membrane microfiltration and ultrafiltration (1 kDa molecular weight cutoff membrane) to isolate low-molecular-weight peptides. The resulting peptide fraction was lyophilized and stored at −20 °C until use. All experiments were conducted in accordance with institutional ethical guidelines. The use of *T. fasciatus* skin was approved by [Fisheries Research Institute of Fujian, FRIF21-2502-05]. All cell lines were authenticated, tested for mycoplasma contamination, and maintained under standard culture conditions.

### 4.2. Identification of Polypeptide Sequences by Nano-Scale High-Performance Liquid Chromatography-Tandem Mass Spectrometry

The peptides were re-dissolved in solvent A (A: 0.1% formic acid in water) and analyzed by Orbitrap Q-Exactive Plus coupled to an EASY-nanoLC 1200 system (Thermo Fisher Scientific, Waltham, MA, USA). A total of 1 μL of sample was loaded (analytical column: Acclaim PepMap C18, 75 μm × 25 cm). Samples were separated in a 60 min gradient, with a controlled column flow rate of 300 nL/min, a column temperature of 40 °C, an electrospray voltage of 2 kV, and a gradient starting at 2% phase B at 47 min. The bell was raised to 35% in a nonlinear gradient and to 100% in 1 min for 12 min. The mass spectrometry parameters were set as follows: (1) MS—Scan range (*m*/*z*): 200–1800; Resolution: 70,000; AGC target: 3 × 10^6^; Maximum injection time: 50 ms; (2) HCD-MS/MS—Resolution: 17,500; AGC target: 1 × 10^5^; Maximum injection time: 45 ms; Collision energy: 28%; Dynamic exclusion time: 30 s.

### 4.3. Virtual Screening of Hit Peptides

Based on the MS results, peptides with a confidence −logp > 20 and molecular weight <1 kDa were selected. Peptides with bioactivity >0.6 were preliminarily screened using the Peptide Ranker program (http://distilldeep.ucd.ie/PeptideRanker/, accessed on 10 April 2025). The 3D structure of TRPV1 (PDB: 8GFA) was retrieved from the RCSB PDB database (https://www.rcsb.org, accessed on 10 April 2025). Receptor preparation was performed using MOE 2022 software (Chemical Computing Group, Montreal, ON, Canada), including the removal of crystallographic water molecules, hydrogen addition, charge assignment, and energy minimization. The peptide ligands were modeled using Discovery Studio 2019 (Dassault Systèmes, Paris, France). The active site of TRPV1 was defined at coordinates (X: 108.603; Y: 79.704; and Z: 88.011), encompassing the following residues: TYR511, SER512, LEU515, TYR543, ALA546, LEU547, THR550, ASN551, LEU553, TYR554, ARG557, ALA566, ILE569, GLU570, and ILE573. Virtual screening was conducted using the DOCK 6.9 program with molecular docking-based protocols. The top 20 hit peptides were prioritized based on grid score values and binding mode analysis. These candidate peptides were subsequently synthesized using solid-phase peptide synthesis for experimental validation.

### 4.4. Cell Viability and NO Content Assays

The 20 peptides with the top scores were selected and entrusted to Nanjing GenScript Biotechnology Co., Ltd., with synthetic sample purity > 95%. The process was as follows: Select the appropriate resin, pretreat it with solvent, remove the resin protective group, and expose the active site. The first protective amino acid is activated and coupled to the resin, and the amino protecting group of the amino acid is removed after washing. Repeat the “Activation–Conjugation–Deprotection” step and gradually add amino acids to lengthen the peptide chain. The peptide is cleaved from the resin with reagents such as TFA while the side-chain guard group is removed. Peptides are precipitated, washed, purified by HPLC, and identified by mass spectrometry. RAW 264.7 cells were purchased from Peking Union Medical College. The cytotoxicity of 20 candidate peptides towards RAW 264.7 cells is evaluated in an MTS assay (Promega, Madison, WI, USA), adapted from reference [44] with modifications. RAW 264.7 cells are maintained in Dulbecco’s Modified Eagle Medium (DMEM, C11995500BT, Gibco, Grand Island, NY, USA) supplemented with 10% fetal bovine serum (F8687, Merck, Darmstadt, Germany) and 1% penicillin-streptomycin (C0222, Beyotime, Shanghai, China) at 37 °C under 5% CO_2_. Cells in the logarithmic growth phase are harvested, resuspended at 1 × 10^5^ colony-forming units (CFU)/mL, and seeded into 96-well plates (100 μL/well). After incubation for 8–12 h, the supernatant is aspirated, and cells are treated with 100 μL peptide solution (200 μM) for 2 h. LPS (L8880, Solarbio) is then added (100 μL/well, 1 μg/mL) and incubated for an additional 24 h. NO production is measured using a NO detection kit (G2930, Promega) following the manufacturer’s protocol.

### 4.5. Ca^2+^ Content Assay

HaCaT cells were purchased from the Kunming Cell Bank, Chinese Academy of Sciences. Adapted from a previous study [45] with modifications, HaCaT cells were cultured in DMEM supplemented with 10% fetal bovine serum and 1% penicillin-streptomycin at 37 °C under 5% CO_2_. Cells in the logarithmic growth phase were harvested and adjusted to a density of 1 × 10^5^ CFU/mL. Cell suspensions (100 μL/well) were seeded into 96-well plates and incubated for 8–12 h. After removing the supernatant, the cells were treated with T10, T14, or T18 peptide solutions (200 μM) for 2, 6, 12, or 24 h, followed by stimulation with 100 μL CAP (IC0060, Solarbio) per well. Untreated cells served as blank controls. Intracellular Ca^2+^ levels were quantified using a calcium ion assay kit (S1061S; Beyotime) according to the manufacturer’s protocol. Fluorescence images were captured using an inverted fluorescence microscope and analyzed using ImageJ software (NIH, Bethesda, MD, USA) to calculate the average fluorescence intensity.

### 4.6. Mouse Cytokine Array Analysis

The expression levels of 40 cytokines and chemokines in RAW 264.7 cell culture supernatants were analyzed using the Proteome Profiler™ Mouse Cytokine Array Panel A (ARY006, R&D Systems, Minneapolis, MN, USA) following the manufacturer’s protocol. Supernatants from treated groups were collected, and 300 μL per sample was used for each array. All reagents were equilibrated to room temperature (26 ± 0.5 °C) prior to use. The array membrane was blocked with blocking buffer for 2 h at room temperature. During blocking, the detection antibody cocktail was mixed with the cell supernatant and incubated at room temperature for 1 h. The mixture was then transferred to the blocked membrane and incubated overnight at 4 °C. Post-incubation, the membrane was washed three times with wash buffer (≥10 min per wash). Horseradish peroxidase-conjugated streptavidin was added, and the membrane was incubated for 2 h at room temperature. After thorough washing, a chemiluminescent substrate was applied, and signals were captured using a chemiluminescence imaging system. The array images were analyzed using ImageJ software to quantify the integrated optical density.

### 4.7. Western Blot Analysis of NF-κB Pathway Protein Expression

Adapted from a previous study [46] with modifications, HaCaT cells were seeded into 6-well plates at a density of 2.0 × 10^5^ CFU/mL and cultured for 8–12 h. Cells were pretreated with varying concentrations of T14 for 6 h, followed by co-stimulation with CAP for 24 h. After washing with Dulbecco’s phosphate-buffered saline, the cells were lysed on ice for 1 h using RIPA Lysis Buffer (R0010, Solarbio). Lysates were centrifuged at 12,000× *g* for 30 min at 4 °C, and the supernatants were collected. Protein concentrations were quantified using a BCA assay and normalized. Loading buffer (P1016, Solarbio) was added at a 1:3 ratio, and the samples were denatured at 95 °C for 5 min using a heat block before being stored at −20 °C.

Proteins (10 μL/lane) were resolved via sodium dodecyl sulfate-polyacrylamide gel electrophoresis and transferred onto polyvinylidene fluoride membranes. Membranes were blocked with 5% skim milk in Tris-buffered saline containing 0.1% Tween-20 for 2 h at room temperature. Primary antibodies against NF-κB p65 (8242T, Cell Signaling Technology, Danvers, MA, USA) and GAPDH (CL594-6004, Abclonal, Wuhan, China) were diluted according to the manufacturer’s guidelines and incubated overnight at 4 °C. After washing with Tris-buffered saline containing 0.1% Tween-20, the membranes were incubated with horseradish peroxidase-conjugated secondary antibodies for 1 h at room temperature. Protein bands were visualized using ECL Western Blotting Substrate (K-12045-D50, Advansta, San Jose, CA, USA) and imaged using a chemiluminescence detection system. Band intensities were quantified using ImageJ software.

### 4.8. HUVEC Cell Stimulatory Response

HUVEC cell were purchased from Peking Union Medical College. HUVECS were cultured according to protocols provided by the Peking Union Medical College Cell Bank. Cells were maintained in DMEM supplemented with 10% fetal bovine serum, 1% penicillin-streptomycin, 1% GlutaMAX™ (25030149, Gibco), 1% non-essential amino acids (N1250, Solarbio), and 2% sodium pyruvate (11360070, Gibco) at 37 °C under 5% CO_2_. Cells in the logarithmic growth phase were seeded into 24-well plates at 2 × 10^5^ CFU/mL and incubated overnight. After supernatant removal, the cells were pretreated with varying concentrations of T14 peptide (50, 100, 200, 400 μM) for 6 h, followed by co-stimulation with LPS (10 μg/mL) for 24 h. Cell culture supernatants were collected, and the expression levels of ET-1 and ICAM-1 were quantified using ELISA kits (E-EL-H0064 for ET-1, E-EL-H6114 for ICAM-1; Procell, Wuhan, China) according to the manufacturer’s instructions. The inhibition rate was calculated using the following formula:

Inhibition rate (%) = (LPS − peptide)/(LPS − control) × 100%

### 4.9. Network Pharmacology Analysis

#### 4.9.1. Prediction of Potential Therapeutic Targets of T14 in SS

Adapted from a previous study [47], the potential biological targets of T14 were predicted using the Swiss Target Prediction platform (http://www.swisstargetprediction.ch/, accessed on 15 April 2025). Gene targets associated with SS were retrieved from the Genecards database (https://www.genecards.org/, accessed on 15 April 2025) using the keyword “sensitive skin”. A Venn diagram was constructed using the JVenn tool (https://www.bioinformatics.com.cn/static/others/jvenn/example.html, accessed on 15 April 2025) to identify overlapping targets between T14 and SS-related genes.

#### 4.9.2. PPI Network Construction

Potential therapeutic targets of the peptide in SS were uploaded to the STRING database (https://cn.string-db.org/, accessed on 15 April 2025), with the organism set to *Homo sapiens* and a confidence score threshold >0.7 to construct a PPI network. The network was visualized and refined using Cytoscape 3.10.0 (Cytoscape Consortium, La Jolla, CA, USA). Topological parameters, including degree centrality—a metric reflecting node importance, where higher values indicate greater network influence—were calculated using the CytoNCA plug-in.

#### 4.9.3. KEGG Pathway Enrichment and GO Analysis

KEGG pathway enrichment analysis and GO analysis were performed to predict the mechanisms underlying the efficacy of the peptide in alleviating SS. Potential therapeutic targets were uploaded to the DAVID platform (https://david.ncifcrf.gov/home.jsp, accessed on 16 April 2025), with the species restricted to *Homo sapiens* and a significance threshold of *p* < 0.05 [48,49]. Enriched pathways and functional terms were visualized using the Weishengxin Platform (http://www.bioinformatics.com.cn/, accessed on 16 April 2025).

### 4.10. Data Processing

Statistical analysis was conducted using GraphPad Prism 8.0.2 (GraphPad, Inc., La Jolla, CA, USA). Data were analyzed using one-way analysis of variance followed by Dunnett’s multiple comparison tests, with *p* < 0.05 considered to indicate statistically significant results.

## 5. Conclusions

This study identified, for the first time, a naturally derived peptide (T14) with TRPV1-inhibitory activity from the processing byproducts of *T*. *fasciatus* skin through molecular docking and in vitro cellular models. We elucidated its multi-mechanistic role in alleviating skin sensitivity symptoms by modulating TRPV1 receptor function, suppressing the NF-κB inflammatory signaling pathway, and improving cutaneous microvascular hyperreactivity. Network pharmacology further revealed that T14 may regulate neuro-immune crosstalk via targets such as STAT3 and MAPK3, highlighting its multi-target synergistic properties. Compared to synthetic TRPV1 inhibitors, T14, as a fish-derived natural peptide, exhibits the advantages of low allergenicity and high biocompatibility. This work not only provides a novel strategy for valorizing aquatic processing waste but also advances the mechanistic understanding of marine bioactive peptides in regulating skin physiological homeostasis. As a promising drug candidate, future research should prioritize in vivo toxicological profiling, formulation optimization for dermal/transdermal delivery, and clinical validation of its efficacy in neurogenic inflammation models. Furthermore, structural stabilization strategies to enhance protease resistance and bioavailability will be essential for translational development.

## Figures and Tables

**Table 1:** Sequence and docking scores of the 20 prioritized peptides binding to TRPV1.

Number	ID	Grid_Score
T1	WTKIHF	−106.138
T2	FSWLA	−103.8745
T3	LFNW	−95.4542
T4	WSPW	−89.0253
T5	IFDL	−87.6124
T6	SLQFF	−87.3296
T7	WLGY	−86.9512
T8	MQGW	−86.6209
T9	LFIL	−85.6879
T10	QFF	−84.5399
T11	IFSL	−84.5094
T12	GFIF	−84.2106
T13	YDF	−83.7816
T14	LDIF	−83.5353
T15	LRF	−83.4104
T16	PAGGGDPI	−83.3656
T17	RWI	−83.2909
T18	FFR	−82.5188
T19	FID	−82.3704
T20	QMML	−82.144

**Table 2:** Optimal conformational interaction forces between peptides and TRPV1.

Peptides	RMSD (Å)	Residues with Hydrogen Bonds	Residues with Hydrophobic Interactions	Residues with Electrostatic Action	Sulfur
T10	3.27	ASN438 (2.77 Å, 2.69 Å) ARG491 (2.73 Å, 2.82 Å) TYR555 (2.38 Å, 2.12 Å)	VAL441 (5.00 Å) PHE488 (4.71 Å) ARG491 (4.43 Å)	ARG491 (4.52 Å) GLU513 (3.51 Å) TYR554 (4.07 Å)	
T14	2.96	THR550 (2.24 Å)	MET572 (4.26 Å) LEU553 (5.27 Å) TYR511 (4.59 Å) LEU515 (5.32 Å) LEU553 (5.27 Å) LEU577 (4.83 Å, 4.96 Å) ILE569 (4.78 Å)		MET568 (5.86 Å) MET683 (5.95 Å)
T18	3.24	TYR554 (1.95 Å) ASN438 (2.64 Å) ARG491 (4.87 Å) THR709 (2.52 Å)	PHE516 (4.04 Å) TYR554 (4.86 Å) VAL441 (5.28 Å)	ARG491 (4.93 Å) TYR487 (4.36 Å)	

RMSD, root mean square deviation.

**Table 3:** Core targets and topological parameters.

Number	Target	Degree
1	STAT3	24
2	MAPK3	22
3	SPHK1	18
4	CTSB	18
5	HDAC1	16
6	HDAC6	12
7	PLAU	12
8	SERPINE1	12
9	CTSG	10

## Data Availability

Data obtained in this study are available from the corresponding author upon request.

## References

[1] He L. (2024). Chinese Clinical Guidelines for Sensitive Skin (2024 edition). Chin. J. Dermatol. Venereol..

[2] Jourdain R., de Lacharrière O., Bastien P., Maibach H.I. (2002). Ethnic variations in self-perceived sensitive skin: Epidemiological survey. Contact Dermat..

[3] Willis C.M., Shaw S., De Lacharrière O., Baverel M., Reiche L., Jourdain R., Bastien P., Wilkinson J.D. (2001). Sensitive skin: An epidemiological study. Br. J. Dermatol..

[4] Misery L., Morisset S., Seite S., Brenaut E., Ficheux A., Fluhr J.W., Delvigne V., Taieb C. (2021). Relationship between sensitive skin and sleep disorders, fatigue, dust, sweating, food, tobacco consumption or female hormonal changes: Results from a worldwide survey of 10 743 individuals. J. Eur. Acad. Dermatol. Venereol..

[5] Brenaut E., Misery L., Taieb C. (2019). Sensitive Skin in the Indian Population: An Epidemiological Approach. Front. Med..

[6] Jansen C., Shimoda L.M.N., Kawakami J.K., Ang L., Bacani A.J., Baker J.D., Badowski C., Speck M., Stokes A.J., Small-Howard A.L. (2019). Myrcene and terpene regulation of TRPV1. Channels.

[7] Wei X. (2023). Study on Virtual Screening for Targeted TRPV1 Channel Modulators. Master’s Thesis.

[8] Li D.G., Du H.Y., Gerhard S., Imke M., Liu W. (2017). Inhibition of TRPV1 prevented skin irritancy induced by phenoxyethanol. A preliminary in vitro and in vivo study. Int. J. Cosmet. Sci..

[9] Zhou K.S. (2021). Research progress of TRPV1 channel in pathologic pain. Chin. J. Pain Med..

[10] Pan H., Wang Y. (2018). Efficacy evaluation of cosmetics (V) Scientific support for soothing skin efficacy claims. China Surfactant Deterg. Cosmet..

[11] Kedzierski R.M., Yanagisawa M. (2001). Endothelin system: The double-edged sword in health and disease. Annu. Rev. Pharmacol. Toxicol..

[12] Teder P., Noble P.W. (2000). A Cytokine Reborn?. Am. J. Respir. Cell Mol. Biol..

[13] Iosageanu A., Ilie D., Craciunescu O., Seciu-Grama A.M., Oancea A., Zarnescu O., Moraru I., Oancea F. (2021). Effect of Fish Bone Bioactive Peptides on Oxidative, Inflammatory and Pigmentation Processes Triggered by UVB Irradiation in Skin Cells. Molecules.

[14] Xiong X., Liang J., Xu Y., Liu J., Liu Y. (2020). The wound healing effects of the Tilapia collagen peptide mixture TY001 in streptozotocin diabetic mice. J. Sci. Food Agric..

[15] Fu Y., Li C.Y., Wang Q., Gao R.C., Cai X., Wang S., Zhang Y.H. (2022). The protective effect of collagen peptides from bigeye tuna (*Thunnus obesus*) skin and bone to attenuate UVB-induced photoaging via MAPK and TGF-β signaling pathways. J. Funct. Foods.

[16] Maia Campos P., Franco R.S.B., Kakuda L., Cadioli G.F., Costa G.D., Bouvret E. (2021). Oral Supplementation with Hydrolyzed Fish Cartilage Improves the Morphological and Structural Characteristics of the Skin: A Double-Blind, Placebo-Controlled Clinical Study. Molecules.

[17] Chen B., Zhang G., Qiao K., Xu M., Cai S., Liao D., Liu Z. (2020). Preparation of collagen peptide fromFugu bimaculatusskin and its efficacy and irritation evaluation in cosmetics. Nat. Prod. Res. Dev..

[18] Chen B., Xue K., Tang H., Zhang G., Qiao K., Xu M., Zhang D., Liu Z. (2024). Photoprotective effect of collagen peptides from the skin of Takifugu Photoprotective effect of collagen peptides from the skin of Takifugu. J. Fish. Res..

[19] Zhang G. (2018). Extaction of Collagen Peptides from fugu Bimacujatus Skin and Their Cosmeticefficacy. Master’s Thesis.

[20] Sharma J.N., Al-Omran A., Parvathy S.S. (2008). Role of nitric oxide in inflammatory diseases. Inflammopharmacology.

[21] Hou C., Nie C., Wang Y., Ai L., Xia Y., Zhang H., Xie F., Wang G. (2021). Rapid Screening and Verification of α-Lactalbumin-Derived ACE Inhibitory Peptides. Food Sci..

[22] Pittayapruek P., Meephansan J., Prapapan O., Komine M., Ohtsuki M. (2016). Role of Matrix Metalloproteinases in Photoaging and Photocarcinogenesis. Int. J. Mol Sci..

[23] Cui A., Shao Y., Song J., Wang Y. (2024). Advancements in research on immune cells and cytokines in the treatment of acute lung injury. Proceeding Clin. Med..

[24] Hou Z., Huang Y., Shi X., Zhou Y., Zhang Y. (2023). Research progress on genetically engineered cytokines. Chin. J. Biol..

[25] Nakahara T., Kido-Nakahara M., Ulzii D., Miake S., Fujishima K., Sakai S., Chiba T., Tsuji G., Furue M. (2020). Topical application of endothelin receptor a antagonist attenuates imiquimod-induced psoriasiform skin inflammation. Sci. Rep..

[26] Singh M., Thakur M., Mishra M., Yadav M., Vibhuti R., Menon A.M., Nagda G., Dwivedi V.P., Dakal T.C., Yadav V. (2021). Gene regulation of intracellular adhesion molecule-1 (ICAM-1): A molecule with multiple functions. Immunol. Lett..

[27] Qian H., Peng Y., Huang X., Yu S., Liu B., Wang N., Zhao Y. (2021). Mechanism of Anti-depression Mechanism of Akebiae Fructus Basedon Network Pharmacology. Sci. Technol. Food Ind..

[28] Yang F., Zheng J. (2017). Understand spiciness: Mechanism of TRPV1 channel activation by capsaicin. Protein Cell.

[29] Becher B., Tugues S., Greter M. (2016). GM-CSF: From Growth Factor to Central Mediator of Tissue Inflammation. Immunity.

[30] Napetschnig J., Wu H. (2013). Molecular basis of NF-κB signaling. Annu. Rev. Biophys..

[31] Cheong R., Hoffmann A., Levchenko A. (2008). Understanding NF-κB signaling via mathematical modeling. Mol. Syst. Biol..

[32] Maurer M., Stebut E. (2004). Macrophage inflammatory protein-1. Int. J. Biochem. Cell Biol..

[33] Lin J., Xu Y., Guo P., Chen Y., Zhou J., Xia M., Tan B., Liu X., Feng H., Chen Y. (2023). CCL5/CCR5-mediated peripheral inflammation exacerbates blood–brain barrier disruption after intracerebral hemorrhage in mice. J. Transl. Med..

[34] Fernandes S.E., Saini D.K. (2021). The ERK-p38MAPK-STAT3 Signalling Axis Regulates iNOS Expression and Salmonella Infection in Senescent Cells. Front. Cell. Infect. Microbiol..

[35] GONG T., Si K., Liu H., Zhang X. (2022). Research advances in the role of MAPK cascade in regulation of cell growth, immunity, inflammation, and cancer. J. Cent. South Univ. (Med. Sci.).

[36] DeVore S.B., Schuetz M., Alvey L., Lujan H., Ochayon D.E., Williams L., Chang W., Filuta A., Ruff B., Kothari A. (2024). Regulation of MYC by CARD14 in human epithelium is a determinant of epidermal homeostasis and disease. Cell Rep..

[37] Carmeliet P. (2005). Angiogenesis in life, disease and medicine. Nature.

[38] Krizanova O., Penesova A., Sokol J., Hokynkova A., Samadian A., Babula P. (2022). Signaling pathways in cutaneous wound healing. Front. Physiol..

[39] Bodó E., Kovács I., Telek A., Varga A., Paus R., Kovács L., Bíró T. (2004). Vanilloid receptor-1 (VR1) is widely expressed on various epithelial and mesenchymal cell types of human skin. J. Investig. Dermatol..

[40] Ghorbanzadeh S., Khojini J.Y., Abouali R., Alimardan S., Zahedi M., Tahershamsi Z., Tajbakhsh A., Gheibihayat S.M. (2024). Clearing the Path: Exploring Apoptotic Cell Clearance in Inflammatory and Autoimmune Disorders for Therapeutic Advancements. Mol. Biotechnol..

[41] Talagas M., Misery L. (2019). Role of Keratinocytes in Sensitive Skin. Front. Med..

[42] Zhu N., Gu L., Jia J., Wang X., Wang L., Yang M., Yuan W. (2018). Endothelin-1 triggers human peritoneal mesothelial cells’ proliferation via ERK1/2-Ets-1 signaling pathway and contributes to endothelial cell angiogenesis. J. Cell. Biochem..

[43] Shen S., Fan J., Cai B., Lv Y., Zeng M., Hao Y., Giancotti F.G., Fu B.M. (2009). Vascular endothelial growth factor enhances cancer cell adhesion to microvascular endothelium in vivo. Exp. Physiol..

[44] Yao Y., Liu K., Zhao Y., Hu X., Wang M. (2018). Pterostilbene and 4′-Methoxyresveratrol Inhibited Lipopolysaccharide-Induced Inflammatory Response in RAW264.7 Macrophages. Molecules.

[45] Xu R.Q., Ma L., Chen T., Zhang W.X., Chang K., Wang J. (2023). Sophorolipid inhibits histamine-induced itch by decreasing PLC/IP3R signaling pathway activation and modulating TRPV1 activity. Sci. Rep..

[46] Zheng J., Zhang R.J., Chen Y.M., Ye X., Chen Q.X., Shen D.Y., Wang Q. (2017). Synthesis of caffeic acid ester morpholines and their activation effects on tyrosinase. Process Biochem..

[47] Xie Y., Ge W., Li G., Bai H., Zhang J., Li X., Gao Q., Wang S. (2023). In Silico Analysis of Novel DPP-IV Inhibitory Peptides Released from Camel Milk Lactoferrin and the Possible Pathways Involved in Diabetes Protection. Sci. Technol. Food Ind..

[48] Zhang L., Han L., Ma J., Wu T., Wei Y., Zhao L., Tong X. (2022). Exploring the synergistic and complementary effects of berberine and paeoniflorin in the treatment of type 2 diabetes mellitus by network pharmacology. Eur. J. Pharmacol..

[49] Zhao L., Zhang M., Pan F., Li J., Dou R., Wang X., Wang Y., He Y., Wang S., Cai S. (2021). In silico analysis of novel dipeptidyl peptidase-IV inhibitory peptides released from Macadamia integrifolia antimicrobial protein 2 (MiAMP2) and the possible pathways involved in diabetes protection. Curr. Res. Food Sci..

